# Clustering of Health Behaviors in Canadians: A Multiple Behavior Analysis of Data from the Canadian Longitudinal Study on Aging

**DOI:** 10.1093/abm/kaad008

**Published:** 2023-05-08

**Authors:** Zack van Allen, Simon L Bacon, Paquito Bernard, Heather Brown, Sophie Desroches, Monika Kastner, Kim L Lavoie, Marta M Marques, Nicola McCleary, Sharon Straus, Monica Taljaard, Kednapa Thavorn, Jennifer R Tomasone, Justin Presseau

**Affiliations:** Clinical Epidemiology Program, Ottawa Hospital Research Institute, Ottawa, ON, Canada; School of Psychology, University of Ottawa, Ottawa, ON, Canada; Department of Health, Kinesiology, and Applied Physiology, Concordia University, Montreal, QC, Canada; Montreal Behavioural Medicine Centre, CIUSSS‐NIM, Montreal, QC, Canada; Department of Physical Activity Sciences, University of Quebec at Montreal, Montreal, QC, Canada; Research Center of the Montreal Mental Health University Institute, Montreal, QC, Canada; Lancaster University, Division of Health Research, LancasterUK; School of Nutrition, Laval University, Quebec City, QC, Canada; North York General Hospital, Toronto, ON, Canada; Montreal Behavioural Medicine Centre, CIUSSS‐NIM, Montreal, QC, Canada; Departement is Psychology, University of Quebec at Montreal, Montreal, QC, Canada; Comprehensive Health Research Centre (CHRC), NOVA Medical School|Faculdade de Ciências Médicas (NMS|FCM,) Universidade Nova de Lisboa, Lisboa, Portugal; Clinical Epidemiology Program, Ottawa Hospital Research Institute, Ottawa, ON, Canada; School of Epidemiology and Public Health, University of Ottawa, Ottawa, ON, Canada; Knowledge Translation Program, Li Ka Shing Knowledge Institute, St. Michael’s Hospital, Unity Health Toronto, Toronto, Canada; Department of Medicine, University of Toronto, Toronto, ON, Canada; Clinical Epidemiology Program, Ottawa Hospital Research Institute, Ottawa, ON, Canada; School of Epidemiology and Public Health, University of Ottawa, Ottawa, ON, Canada; Clinical Epidemiology Program, Ottawa Hospital Research Institute, Ottawa, ON, Canada; School of Epidemiology and Public Health, University of Ottawa, Ottawa, ON, Canada; School of Kinesiology and Health Studies, Queens University, Kingston, ON, Canada; Clinical Epidemiology Program, Ottawa Hospital Research Institute, Ottawa, ON, Canada; School of Psychology, University of Ottawa, Ottawa, ON, Canada; School of Epidemiology and Public Health, University of Ottawa, Ottawa, ON, Canada

**Keywords:** Health behaviors, Multiple behaviors, Cluster analysis, CLSA

## Abstract

**Background:**

Health behaviors such as physical inactivity, unhealthy eating, smoking tobacco, and alcohol use are each leading risk factors for non-communicable chronic disease. Better understanding which behaviors tend to co-occur (i.e., cluster together) and co-vary (i.e., are correlated) may provide novel opportunities to develop more comprehensive interventions to promote multiple health behavior change. However, whether co-occurrence or co-variation-based approaches are better suited for this task remains relatively unknown.

**Purpose:**

To compare the utility of co-occurrence vs. co-variation-based approaches for understanding the interconnectedness between multiple health-impacting behaviors.

**Methods:**

Using baseline and follow-up data (*N* = 40,268) from the Canadian Longitudinal Study of Aging, we examined the co-occurrence and co-variation of health behaviors. We used cluster analysis to group individuals based on their behavioral tendencies across multiple behaviors and to examine how these clusters are associated with demographic characteristics and health indicators. We compared outputs from cluster analysis to behavioral correlations and compared regression analyses of clusters and individual behaviors predicting future health outcomes.

**Results:**

Seven clusters were identified, with clusters differentiated by six of the seven health behaviors included in the analysis. Sociodemographic characteristics varied across several clusters. Correlations between behaviors were generally small. In regression analyses individual behaviors accounted for more variance in health outcomes than clusters.

**Conclusions:**

Co-occurrence-based approaches may be more suitable for identifying sub-groups for intervention targeting while co-variation approaches are more suitable for building an understanding of the relationships between health behaviors.

## Teaser

Individual health behaviours (e.g., physical inactivity, unhealthy eating, smoking tobacco, alcohol) were stronger predictors of future health outcomes than were groupings of people based on patterns of co-occurring health behaviours.

Non-communicable chronic diseases such as chronic respiratory disease, diabetes, cardiovascular disease, and cancer cause two-thirds of annual deaths in Canada and worldwide [1–3]. Furthermore, nearly 12% of people aged 65 or older have lived with two or more chronic conditions during their lifetime [4]. Health behaviors such as smoking, excessive alcohol consumption, physical inactivity, and unhealthy eating are strongly associated with quality of life and are leading risk factors for chronic diseases [5]. With approximately four in five adult Canadians engaging in at least one of the health-impacting behaviors associated with non-communicable chronic diseases, the prevalence of risky health behaviors is high [4].

The consequences and risk factors of multimorbidity (living with two or more chronic conditions) has been studied extensively [6–8]; however, research seeking to understand the relationships between life satisfaction, general health, and different combinations of health behaviors has received comparably little attention. Our daily lives are characterized by multiple interconnected social, personal, family, health, and work-related behaviors, each contesting for the limited energy, motivation, and time available [9]. Despite this, health risk behaviors are generally promoted and studied in isolation resulting in interventions and guidelines for healthy living siloed by individual behaviors. For example, historically Canada has had separate guidelines for alcohol consumption [10, 11], and physical activity and sleep [12], although recent guidelines are beginning to incorporate multiple health behaviors (e.g., guidelines for movement behaviors including sleep, sedentary activity, and physical activity [13]). The move towards guidelines that cover multiple health behaviors provide an opportunity to develop an evidence base to reflect an understanding of which health behaviors are interconnected, and how these patterns of interconnectedness are associated with health care utilization, life satisfaction, physical health, and mental health. This in turn may provide new opportunities to promote multiple health behavior change in guidelines and beyond. Indeed, interventions could be tailored to reflect the real-world complexities of health behaviors through an understanding of which behaviors are interconnected and for whom.

When investigating the interconnectedness of multiple health behaviors there are two general approaches: *person-centered approaches* which assess co-occurrence of behaviors and group people into categories, and *variable-centered approaches* which assess co-variation of behaviors through the strength and direction of relationships between behaviors. Person-centered approaches include but are not limited to agglomerative cluster analysis, k-means, latent class analysis, behavioral profiles, and Gaussian mixture models. Applied to multiple health behaviors, person-centered approaches aim to segment people into categories based on similarity of behavioral features to identify focused intervention targets (behavioral combinations) and the sociodemographic patterns associated with each group [14–17].

However, to date research in this area often assesses different combinations of behaviors with heterogeneous measurements which result a wide array of behavioral clusters [14–17]. For example, Conry et al. [14] investigated the clustering of alcohol use, physical activity, smoking, and unhealthy eating in a sample of Irish adults obtained from the 2007 National Survey of Lifestyle, Attitudes, and Nutrition. Six clusters were identified in this cross-sectional analysis which were labeled as: (a) “*multiple risk factor*” (moderate physical activity, moderate to high alcohol use, variable healthy eating); (b) “*mixed lifestyle*” (those who had never smoked, reported moderate physical activity, and variable alcohol consumption); (c) “*physically inactive”* (people with low levels of physical activity, poor eating, who reporting some smoking, and high alcohol use); (d) “*temperate*” (moderately active and moderate drinkers who had never smoked); (e) “*former smokers”* (former smokers who reported high physical activity, moderate alcohol use, and healthy eating); and (f) “*healthy lifestyle”* (characterized by people who had never smoked, high physical activity, highest healthy eating, moderate alcohol use). In another example, Buck and Frosini [15] examined the clustering of unhealthy eating, alcohol use, smoking, and physical inactivity among adults aged 16–74 using 2003–2008 data from the Health Survey of England. Findings indicated that in 2008, 63% of the sample engaged in one or two unhealthy behaviors, 25% engaged in three or more risky health behaviors, and 5% reported engaging in all four measured health behaviors. Only 7% of the sample did not engage in any measured risky health behaviors. Finally, in contrast to these data-driven approached for clustering, Shaw and Agahi [18] used a descriptive approach called “health behaviour profiles” to assess all possible combinations of co-occurring health risk behaviors in American adults 50 years or older using baseline data from the Health and Retirement study [19]. Overall, 12 health behavior profiles were created using all combinations of physically active vs. inactive, smokers vs. nonsmokers, and those who reported no vs. moderate vs. heavy alcohol consumption. The percentage of people represented in each profile varied widely with the six most prevalent profiles including: (a) “physically active, nondrinkers, who smoke” (4.2%); (b) “physically inactive, nondrinkers, who smoke” (6.5%); (c) “physically inactive, moderate drinkers, who do not smoke” (8.6%); (d) “physically active, moderate drinkers, who do not smoke” (10.1%); (e) ‘physically active, nondrinkers, who do not smoke (23.7%); and (f) “physically inactive, nondrinkers, who do not smoke” (34.1%).

The second approach for modeling the relationships between health behaviors are *variable-centered approaches*. The purpose of these types of analyses is to identify the associations between health behaviors to determine the strength and direction of the (usually linear) associations. Examples of variable-centered approaches include (but are not limited to) correlations, multiple regression, network psychometrics, structural equation modeling, and lag-1 temporal time series analysis. The variable-centered approach can help to identify important associations between health behaviors such as the strong positive relationship between healthy eating and exercise [20]. Additionally, revealing the absence of linear relationships, as is the case with the relative independence of sedentary behaviors and physical activity [21], can inform research, policy, and interventions to consider these behaviors as independent from one another.

Although the person- and variable-centered approaches are used in the multiple health behavior literature, to our knowledge there have been no direct comparisons between them. It remains unknown whether person- and variable-centered approaches produce complimentary or divergent insights in the context of multiple health behaviors. It is also unknown whether person or variable analysis is more suitable for understanding the relationships between behaviors and health outcomes (e.g., life satisfaction and general health, onset of chronic conditions, BMI). To this end, we analyzed baseline and follow-up data from the Canadian Longitudinal Study on Aging (CLSA [22]), to (a) identify patterns of co-occurring and co-varying behaviors and assess how sociodemographic and health indicators are associated with these patterns; (b) compare outputs from these two methods; and (c) compare the ability of clusters vs. individual behaviors to predict future health indicators.

## Methods

CLSA is a longitudinal, nationally representative study designed to measure societal, biological, physical, and psychosocial factors related to healthy aging [22]. Baseline data collection for the CLSA was collected between 2010 and 2015 comprising two approaches. First, the “tracking” cohort (*n* = 21,241) completed data collected via an hour-long computer assisted phone interviews. Second, the “comprehensive” cohort (*n* = 30,097) completed an in-person interview lasting 90-min as well as a data collection site visit. Additionally, a “maintaining contact questionnaire” was administered over the phone for the comprehensive and tracking cohorts. The maintaining contact questionnaire, tracking cohort, and comprehensive cohort form the baseline data collected used in this analysis. Detailed methodological information is shown in the published protocol [23]. With follow-up CLSA data subsequently made available, additional analysis was also performed using follow-up data. In the follow-up wave of data collection participants again completed the “tracking” cohort (*n* = 17,050) and the “comprehensive” cohort (*n* = 27,765) packages. A total of *n* = 6,523 participants who completed baseline data collection did not provide follow-up data. Data are available from the Canadian Longitudinal Study on Aging (www.clsa-elcv.ca) for researchers who meet the criteria for access to de-identified CLSA data.

### Participants

Participants were recruited through random-digit dialing, provincial health registries, and the Canadian Community Health Survey on Healthy Aging [22, 24]. Exclusion criteria for the CLSA included: residents living in three territories and First Nations reserves, full time members of the Canadian Armed Forces, people living with cognitive impairments, and individuals living in institutions (including 24-hr nursing homes [22]). Participants included in the study were *n* = 51,338 French and English-speaking Canadians (51% female) between the ages of 45 and 85 at time of enrollment. The average participant age is 62.98 years (*SD* = 10.4) with 26% between 45 and 54 years, 32% between 55 and 64 years, 23% between 65 and74 years, and 18% between 75 and 85 years of age. A full description of demographic characteristics of the sample, as well as summary data across all measured variables is available in the CLSA baseline data report [25]. A total of *n* = 6,523 participants who completed baseline data collection did not provide follow-up data. Thus, we have a sample size of *n* = 44,815 participants who completed follow-up data collection.

### Variables

#### Health behaviors

*Physical activity* and *sedentary behavior* were measured as independent behaviors with the Physical Activity Scale for the Elderly (PASE [26]) which assesses the frequency of sedentary behavior, walking, light physical activity, moderate physical activity, strenuous physical activity, and exercise. Items asked participants to report on their activity levels over the previous 7 days on a 1 (never) to 4 (often, 5–7 days) scale. A Statistics Canada report focusing on the relationship between physical activity and lung functioning [27] merged light and moderate physical activity together and also merged strenuous physical activity and exercise together based on issues with question prompts and conceptual overlap between question items. To facilitate dimension reduction, we opted for a similar approach in which the PASE subscale items were merged to represent: sitting, walking, light/moderate physical activity (renamed “light sports” to avoid confusion with “light-to-moderate physical activity”; e.g., [28]), and strenuous physical activity/ exercise. *Fruit and vegetable consumption* was assessed with one item from the Seniors in the Community Risk Evaluation for Eating and Nutrition questionnaire [29]. The item asks respondents how many servings of fruits and vegetables they eat in a day. The original scale was scored 1 (seven or more) to 7 (less than two); however, items were reverse coded such that higher scores indicate more fruit and vegetable consumption. *Smoking behavior* was measured using a skip-question framework in the CLSA. We assigned a value of 0 to each respondent who responded “no” to the question “have you ever smoked a whole cigarette.” A similar approach has been applied to skip structure data when missing data represent the absence of a behavior or psychological feature [30]. Participants who answered “yes” to the question “have you ever smoked a whole cigarette” were subsequently asked whether they smoke not at all, occasionally, or daily, in the past 30 days. Ultimately, this created four levels distinguishing between people who have never smoked (coded 0), those who have not smoked within 30 days (1), those who smoke occasionally (2), and those who smoke daily (3). *Alcohol use* was assessed with a single item asking participants how often they drank alcohol in the past 12 months on a scale from 1 (almost every day) to 7 (less than once a week). Responses were reverse coded so that higher values indicate greater alcohol consumption. Finally, *sleep* was measured with a single item. Participants were asked how many hours of sleep they get, on average, during the past month and could respond with any value between 0 and 24. This variable was originally included in the analysis plan [23] but was subsequently removed due to high (41.5%) prevalence of missingness.

#### Sociodemographic indicators

We included age, as grouped in the CLSA dataset (45–54; 55–64; 65–74; 75–85), sex (male/female), marital status (single, married or common-law, widowed, divorced, separated), household income (<$20k, $20–$49k, $50–$99k, $100–$149k, $150k+), retirement status (completely retired, partly retired, not retired), and working status (yes/no to “are you currently working at a job or business”).

#### Social support

Participants responded to 19 questions from the Medical Outcomes Study (MOS) Social Support Survey [31]. The MOS is scored on five subscales: tangible social support, affection, positive social interaction, and emotional and informational support. A MOS “overall support index” is also scored in the CLSA baseline dataset. To reduce the number of constructs in our analyses, we used the overall support index, scored from 0 (low support available) to 100 (high support available).

#### General health and life satisfaction

Three single item measures were selected from the CLSA’s general health module: an indicator of general health (“in general, would you say your health is excellent, very good, good, fair, or poor?”), mental health (“in general, would you say your mental health is excellent, very good, good, fair, or poor?”), and perceptions of healthy aging (“in terms of your own healthy aging, would you say it is excellent, very good, good, fair, or poor?”). Items were originally scored on a 1 (excellent) to 5 (poor) but were reverse coded. Additionally, a composite score from the Satisfaction with Life Questionnaire (SWLS [32]) was used. The SWLS is scored according to Diener [33] from 1 (extremely dissatisfied) to 7 (extremely satisfied). A measure of body mass index (BMI) was used as an indicator of physical health.

#### Chronic conditions

Participants reported any diagnosed chronic conditions during baseline and follow-up data collection (e.g., chronic respiratory disease, diabetes, cardiovascular disease, cancer). A summary variable which classifies people into those living with at least one chronic condition at follow-up (1) and those not living with any chronic conditions (0) was used as an indicator of health.

### Analysis

#### Cluster analysis

Prior to performing cluster analysis, all health behavior variables (walking, sitting, light sports, exercise, smoking, alcohol) were standardized (i.e., mean centered) using the scale function in base R [34]. Listwise deletion was applied to missing data in health behavior variables resulting in a remaining total sample of *n* = 40,268 for baseline behaviors. We then performed hierarchical agglomerative cluster analysis with five linkage methods (e.g., complete-linkage, single-linkage, average-linkage, centroid-linkage, and Ward’s method) using “hclust” function supported by the package “fastcluster” [35] to optimize performance. Gower distance was computed using the “daisy” function in the “cluster” package [36].

We examined cluster analysis outputs by looking at summary statistics for health behavior variables for each linkage method. Two of the five linkage methods produced interpretable and useful clustering solutions (i.e., Ward and complete-linkage) while the other methods resulted in clusters with nearly all participants forming a single cluster with a small number of participants (often one per cluster) forming the remaining groups. Next, we employed a data-driven approach to determine the optimal number of clusters using complete-linkage and Ward’s method. We used the NbClust package [37] to provide the top three clustering solutions for both linkage methods, resulting in six options for combinations of linkage measures and *k.* Four of six options produced clustering solutions with 2–3 clusters with minimal variability across behaviors. Of the remaining two options (Ward *k* = 4 and *k* = 7) the research team opted for the clustering solution with seven clusters as this option produced more behavioral variability (i.e., more clusters defined by higher/lower scores on a given behavior).

#### Multinomial logistic regressions (baseline)

We conducted four multinomial logistic regressions predicting cluster membership with baseline data to determine whether clusters are associated with (a) sociodemographic factors, (b) indicators of physical and mental health, (c) non-health behaviors, and (d) health care utilization. Analysis was performed using the “multinom” function from the “nnet” package [38]. Results are presented in Supplementary Appendix II.

### Comparing Person and Variable Approaches

Comparisons between person- and variable-based approaches were conducted in two ways. First, baseline behaviors associated with one another via clusters are descriptively compared with associations assessed with partial polychoric correlations. Partial polychoric correlations (ρ) were computed using the same baseline (*n*= 40,268) sample used for cluster analysis and visualized as a network (Fig. 2). Polychoric correlations are appropriate for ordered categorical data [39–44]. Second, individual health behaviors and clusters were used as predictors in separate regression analyses to predict health outcomes (general health, healthy aging, and the presence of chronic conditions). Ordinary Least Squares (OLS) regression was used to predict general health and mental health while logistic regression was used to predict the presence of chronic conditions. The reference group for the regression analysis was Cluster 4 (“frequent alcohol use and infrequent walkers”) due to most of that cluster’s health behaviors being close to the final sample average. Variance explained (*R*^2^) values are used to compare OLS models while *AIC* is used to compare model fit for logistic regression. No covariates were included in either model.

## Results

### Descriptive Analysis of Clusters

**Fig. 1.**
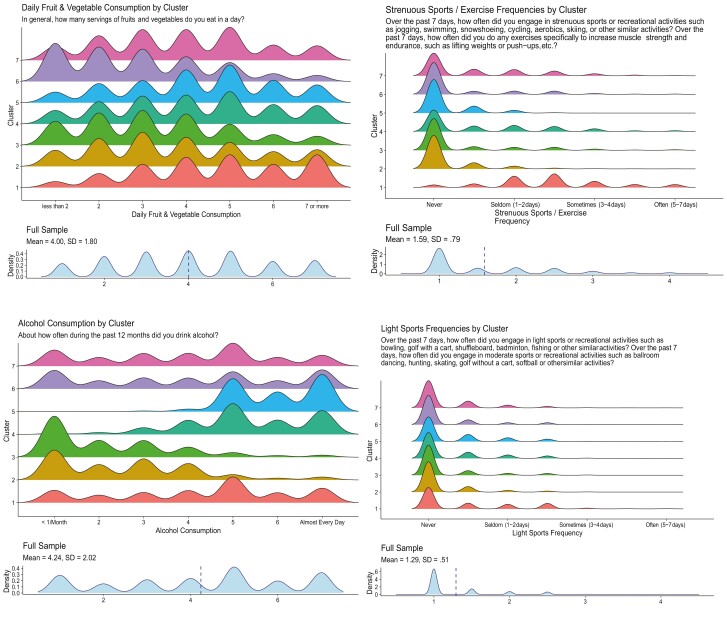

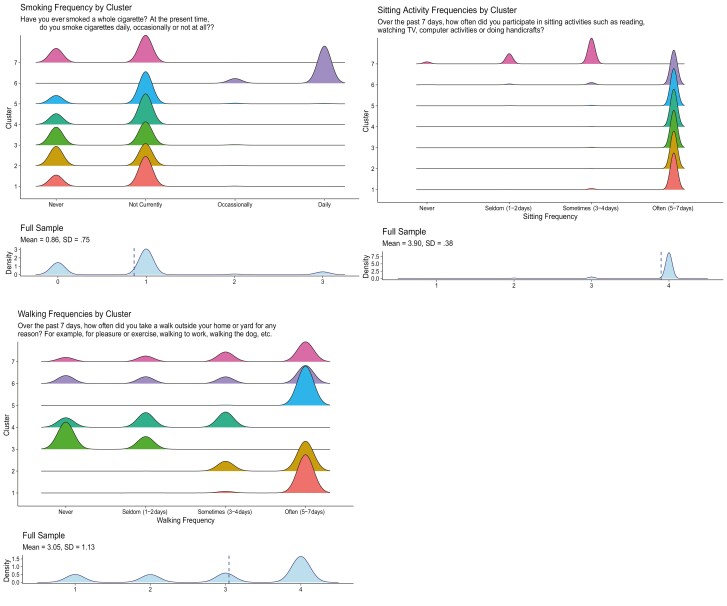
Health behavior ridge (density) plots across clusters. Ridge (density) plots for each health behavior across clusters. *Note*: Cluster labels are as follows: Cluster 1 = physically active healthy eaters; Cluster 2 = frequent walkers with infrequent strenuous exercise and infrequent alcohol use; Cluster 3 = infrequent alcohol use, walking, light sports, and exercise; Cluster 4 = frequent alcohol use and infrequent walkers; Cluster 5 = frequent walkers with infrequent strenuous exercise but higher alcohol use; Cluster 6 = occasional and daily smokers who infrequently eat fruits and vegetables and exercise; Cluster 7 = infrequent sedentary activities

**Fig. 2.**
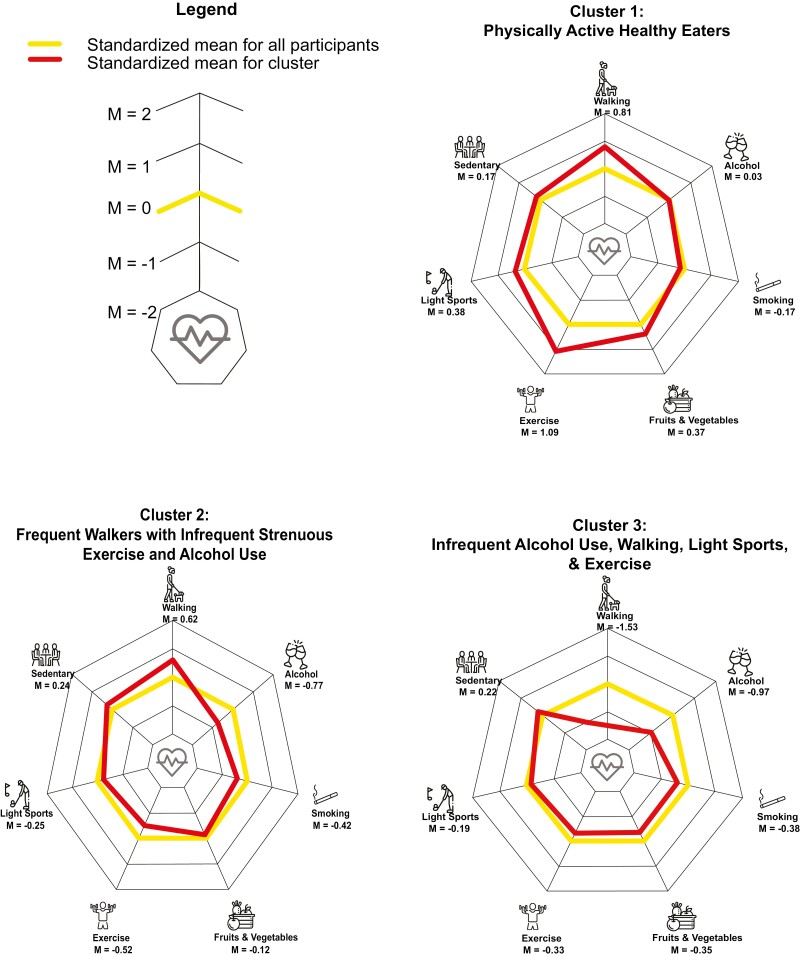

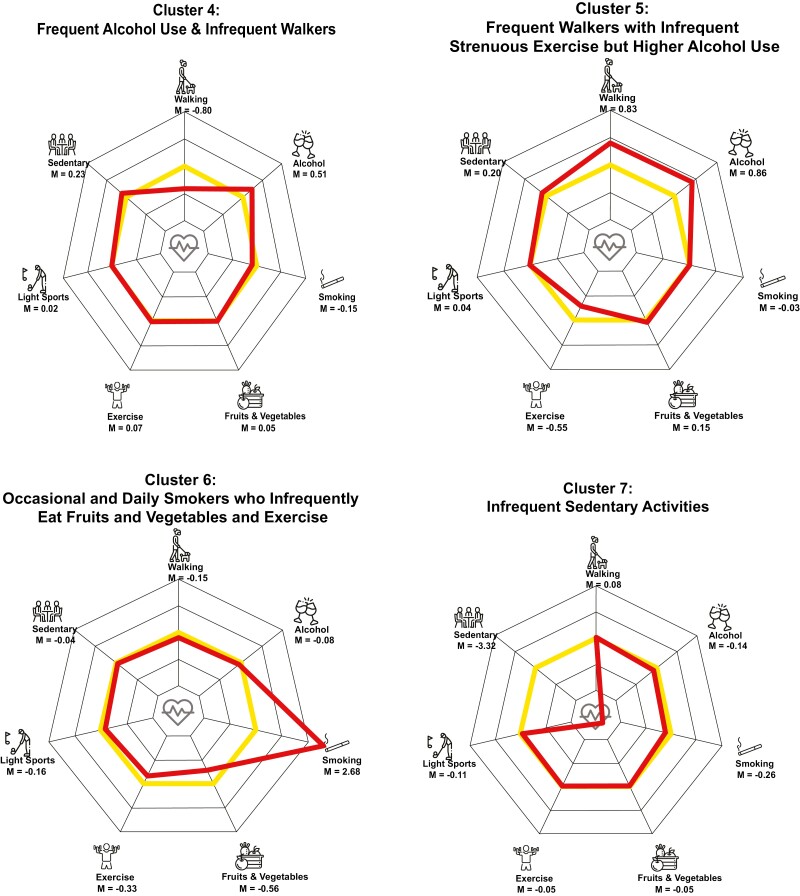
Radar plots for each cluster (standardized means). Radar plots for each cluster (standardized means)

Standardized means and standard deviations for health behaviors in each cluster are presented in Table 1. Demographic information for each cluster is presented in Table 2. Ridge plots illustrating the density distributions of responses for each health behavior across clusters are presented in Fig. 1. Descriptive summaries highlighting the characteristics of the final sample and each cluster, interpreted using unstandardized scales, are provided below and are accompanied by radar charts using standardized scales in Fig. 2.

**Table 1:** Standardized means and standard deviations for baseline health behaviors and clusters

Cluster	n	Walking				Sitting				Light sports				Exercise				Fruit and vegetables				Smoking				Alcohol			
		*M* ^a^	*SD* ^a^	M^b^	*SD* ^b^	*M* ^a^	*SD* ^a^	*M* ^b^	*SD* ^b^	*M* ^a^	*SD* ^a^	*M* ^b^	*SD* ^b^	*M* ^a^	*SD* ^a^	*M* ^b^	*SD* ^b^	*M* ^a^	*SD* ^a^	*M* ^b^	*SD* ^b^	*M* ^a^	*SD* ^a^	*M* ^b^	*SD* ^b^	*M* ^a^	*SD* ^a^	*M* ^b^	*SD* ^b^
1	7703	0.81	0.18	3.96	0.2	0.17	0.53	3.96	0.20	0.38	1.28	1.49	0.65	1.09	0.94	2.44	0.73	0.37	0.96	4.67	1.72	−0.17	0.62	0.74	0.46	0.03	0.95	4.29	1.92
2	7223	0.62	0.38	3.75	0.43	0.24	0.25	3.99	0.10	−0.25	0.71	1.16	0.36	−0.52	0.49	1.18	0.38	−0.12	0.99	3.79	1.78	−0.42	0.68	0.54	0.51	−0.77	0.77	2.67	1.56
3	4094	−1.53	0.41	1.32	0.47	0.22	0.34	3.98	0.13	−0.19	0.83	1.20	0.43	−0.33	0.82	1.33	0.64	−0.35	0.93	3.38	1.68	−0.38	0.68	0.58	0.51	−0.97	0.75	2.29	1.51
4	10723	−0.80	0.69	2.14	0.78	0.23	0.32	3.99	0.12	0.02	1.00	1.30	0.51	0.07	1.01	1.64	0.80	0.05	0.99	4.08	1.78	−0.15	0.60	0.75	0.45	0.51	0.69	5.26	1.39
5	5212	0.83	0.11	3.99	0.12	0.20	0.47	3.98	0.18	0.04	0.96	1.31	0.49	−0.55	0.40	1.16	0.31	0.15	0.95	4.27	1.70	−0.03	0.65	0.84	0.49	0.86	0.48	5.97	0.97
6	3079	−0.15	1.05	2.88	1.19	−0.04	1.07	3.88	0.41	−0.16	0.87	1.21	0.44	−0.33	0.80	1.33	0.63	−0.56	0.93	3.00	1.68	2.68	0.42	2.89	0.32	−0.08	1.07	4.08	2.15
7	2234	0.08	0.91	3.14	1.03	−3.32	1.52	2.62	0.58	−0.11	0.84	1.23	0.43	−0.05	0.90	1.55	0.71	−0.05	0.95	3.91	1.71	−0.26	0.65	0.67	0.49	−0.14	0.96	3.94	1.93
Full Sample	40268	0	1.00	3.05	1.13	0	1.00	3.9	0.38	0	1.00	1.29	0.51	0	1.00	1.59	0.79	0	1.00	4.00	1.80	0	1.00	0.86	0.75	0	1.00	4.24	2.02

^a^Standardized values.

^b^Unstandardized values.

*Notes:* Cluster labels are as follows: Cluster 1 = physically active healthy eaters; Cluster 2 = frequent walkers with infrequent strenuous exercise and infrequent alcohol use; Cluster 3 = infrequent alcohol use, walking, light sports, and exercise; Cluster 4 = frequent alcohol use and infrequent walkers; Cluster 5 = frequent walkers with infrequent strenuous exercise but higher alcohol use; Cluster 6 = occasional and daily smokers who infrequently eat fruits and vegetables and exercise; Cluster 7 = infrequent sedentary activities.

**Table 2:** Counts and percentages of sociodemographic variables for final sample and each cluster

	Full sample		Cluster 1			Cluster 2			Cluster 3			Cluster 4			Cluster 5			Cluster 6			Cluster 7		
	*N*	%	*N*	%	%-%	*N*	%	%-%	*N*	%	%-%	*N*	%	%-%	*N*	%	%-%	*N*	%	%-%	*N*	%	%-%
**Age group**																							
45–54	10923	27.1	2274	29.5	2.4	1852	25.6	−1.5	1013	24.7	−2.4	2760	25.7	−1.4	1040	20	−7.2	1147	37.3	10.1	837	37.5	10.3
55–64	13186	32.7	2613	33.9	1.2	2300	31.8	−0.9	1237	30.2	−2.5	3451	32.2	−0.6	1731	33.2	0.5	1182	38.4	5.6	672	30.1	−2.7
65–74	9461	23.5	1772	23	−0.5	1733	24	0.5	979	23.9	0.4	2591	24.2	0.7	1434	27.5	4	527	17.1	−6.4	425	19	−4.5
75–85	6698	16.6	1044	13.6	−3.1	1338	18.5	1.9	865	21.1	4.5	1921	17.9	1.3	1007	19.3	2.7	223	7.2	−9.4	300	13.4	−3.2
NA	0	0	0	0	0	0	0	0	0	0	0	0	0	0	0	0	0	0	0	0	0	0	0
**Employment status**																							
Yes	16401	40.7	3375	43.8	3.1	2843	39.4	−1.4	1524	37.2	−3.5	4310	40.2	−0.5	1794	34.4	−6.3	1411	45.8	5.1	1144	51.2	10.5
No	1609	4	236	3.1	−0.9	325	4.5	0.5	204	5	1	327	3	−0.9	158	3	−1	267	8.7	4.7	92	4.1	0.1
NA	22258	55.3	4092	53.1	−2.2	4055	56.1	0.9	2366	57.8	2.5	6086	56.8	1.5	3260	62.5	7.3	1401	45.5	−9.8	998	44.7	−10.6
**Sex**																							
Female	20220	50.2	3729	48.4	−1.8	4029	55.8	5.6	2409	58.8	8.6	5025	46.9	−3.4	2310	44.3	−5.9	1563	50.8	0.6	1155	51.7	1.5
Male	20048	49.8	3974	51.6	1.8	3194	44.2	−5.6	1685	41.2	−8.6	5698	53.1	3.4	2902	55.7	5.9	1516	49.2	−0.5	1079	48.3	−1.5
NA	0	0	0	0	0	0	0	0	0	0	0	0	0	0	0	0	0	0	0	0	0	0	0
**Annual income**																							
<$20k	1669	4.1	208	2.7	−1.4	409	5.7	1.5	280	6.8	2.7	229	2.1	-2	122	2.3	−1.8	326	10.6	6.4	95	4.3	0.1
$20–$49k	8663	21.5	1360	17.7	−3.9	1912	26.5	5	1144	27.9	6.4	1973	18.4	−3.1	931	17.9	−3.7	865	28.1	6.6	478	21.4	−0.1
$50–$99k	13928	34.6	2526	32.8	−1.8	2464	34.1	−0.5	1407	34.4	−0.2	3823	35.7	1.1	1941	37.2	2.7	999	32.4	−2.1	768	34.4	−0.2
$100–$149k	7439	18.5	1636	21.2	2.8	1117	15.5	−3	600	14.7	−3.8	2195	20.5	2	1038	19.9	1.4	440	14.3	−4.2	413	18.5	0
$150k+	6214	15.4	1578	20.5	5.1	841	11.6	−3.8	375	9.2	−6.3	1886	17.6	2.2	912	17.5	2.1	260	8.4	−7	362	16.2	0.8
NA	2355	5.8	395	5.1	−0.7	480	6.6	0.8	288	7	1.2	617	5.8	−0.1	268	5.1	−0.7	189	6.1	0.3	118	5.3	−0.6
**Marital status**																							
Single	3230	8	580	7.5	−0.5	685	9.5	1.5	355	8.7	0.7	681	6.4	−1.7	292	5.6	−2.4	465	15.1	7.1	172	7.7	−0.3
Married/CL	28520	70.8	5692	73.9	3.1	4843	67	−3.8	2673	65.3	−5.5	8076	75.3	4.5	3972	76.2	5.4	1697	55.1	−15.7	1567	70.1	−0.7
Widowed	3600	8.9	568	7.4	−1.6	765	10.6	1.7	501	12.2	3.3	868	8.1	−0.8	428	8.2	−0.7	274	8.9	0	196	8.8	−0.2
Divorced	3879	9.6	684	8.9	−0.8	753	10.4	0.8	458	11.2	1.6	871	8.1	−1.5	413	7.9	−1.7	476	15.5	5.8	224	10	0.4
Separated	1027	2.6	175	2.3	−0.3	177	2.5	−0.1	105	2.6	0	225	2.1	−0.5	107	2.1	−0.5	165	5.4	2.8	73	3.3	0.7
NA	12	0	4	0.1	0	0	0	0	2	0	0	2	0	0	0	0	0	2	0.1	0	2	0.1	0.1

*Notes*: %-% denotes the % difference between final sample and a given cluster. Cluster labels are as follows: Cluster 1 = physically active healthy eaters; Cluster 2 = frequent walkers with infrequent strenuous exercise and infrequent alcohol use; Cluster 3 = infrequent alcohol use, walking, light sports, and exercise; Cluster 4 = frequent alcohol use and infrequent walkers; Cluster 5 = frequent walkers with infrequent strenuous exercise but higher alcohol use; Cluster 6 = occasional and daily smokers who infrequently eat fruits and vegetables and exercise; Cluster 7 = infrequent sedentary activities.

#### Final sample

Following listwise deletion for missing values in health behavior variables there were 40,268 people included in the final sample. On average, people engaged in walking activities 3–4 days a week (*M* = 3.1; *SD* = 1.1), sitting activities close to 4–5 days a week (*M* = 3.9; *SD* = 0.4), light sports were mostly performed between “never” and “seldom” (1–2 days per week; *M* = 1.3; *SD* = 0.5), people engaged in strenuous exercise approximately 1 day per week (*M* = 1.6; *SD* = 0.8), ate 4 servings of fruits and vegetables per day (*M* = 4.0; *SD* = 1.8), were nonsmokers (*M* = 0.9; *SD* = 0.8), and consumed alcohol near the middle of a 7 point scale ranging from monthly to daily (*M* = 4.2; *SD* = 2.0). The overall sample was balanced by sex (50.2% Female). The majority were in married or common law relationships (70.8%) with household incomes between $50.000 and $99,000 per year (35.6%). The distribution of age groups was 27.1% (age 45–54), 32.8% (age 55–64), 23.5% (age 65–74), and 16.6% (age 75–85).

#### Cluster 1: Physically active healthy eaters

People assigned to this cluster comprised 19% of all participants and engaged in more walking activities and exercise than the final sample and ate slightly more daily servings of fruits and vegetables. Specifically, people in Cluster 1 engaged in walking activities, on average, closer to 6–7 days a week than the 3–4 days overall average (*M* = 4.0; *SD* = 0.2) and strenuous exercise between 1–2 days a week and 3–4 days a week (*M* = 2.4; *SD* = 0.7). Average daily fruit and vegetable consumption was closer to 5 servings per day (*M* = 4.7; *SD* = 1.7) compared to the overall average of 4 servings per day. When compared to the proportion of people earning $150,000 or more annually in the final sample (15.4%), more people in this cluster earned $150,000 or more (20.5%).

#### Cluster 2: Frequent walkers with infrequent strenuous exercise and infrequent alcohol use

People in this cluster represented 18% of participants and engaged in more frequent walking activities but less frequent strenuous exercise and alcohol consumption when compared to the overall sample. Walking activities were closer to 6–7 days a week than 3–4 days (*M* = 3.8; *SD* = 0.4) while the weekly average for strenuous exercise was closer to “never” than “seldom” (*M* = 1.2; *SD* = 0.4) and alcohol consumption was closer to monthly than daily (*M* = 2.7; *SD* = 1.6). Demographically, there were 5.6% fewer males in this group than the final sample and 5% more people earning $20,000–$49,000 annually.

#### Cluster 3: Infrequent alcohol users, walkers, fruit/vegetable consumption, light sports, and exercise

In this group (10% of participants), all health behaviors were performed less frequently than the group average except for slightly more sitting activities. Notably, the frequencies of walking, light physically activity, and strenuous exercise were each closer to “never” than “seldom (1–2 days)” (*M* = 1.3, 1.2, 1.3; *SD* = 0.5, 0.4, 0.6). Alcohol consumption was lower than average (*M* = 2.3; *SD* = 1.5) indicating that people in this group consumed alcohol closer to monthly than daily. Daily fruit and vegetable servings were closer to 3 servings a week (*M* = 3.4; *SD* = 1.7) than the overall average of 4 servings (*M* = 4.0; *SD* = 1.8). Additionally, the group was comprised of nonsmokers. Demographically, there are more people aged 78–85 in this group (21.1%) compared to overall (16.6%), less Males (41.2%) than overall (49.8%), and the distribution of annual income was skewed towards lower income brackets compared to the final sample with 6.4% more people in Cluster 3 than the final sample earning $20,000–$49,000 and 6.3% less people earning $150,000 per year or more.

#### Cluster 4: Frequent alcohol users and infrequent walkers

The largest of the seven clusters (27%) was defined by near average frequencies of health behaviors with two exceptions. First, the average frequency of walking activities was lower in this cluster with people engaging in walking activities 1–2 days per week (*M* = 2.1; *SD* = 0.8) compared to 3–4 days per week in the final sample (*M* = 3.1; *SD* = 1.1). Second, alcohol consumption was higher (*M* = 5.3; *SD* = 1.4) than the final sample average (*M* = 4.2; *SD* = 2.0) meaning that people in this cluster were closer to daily alcohol consumption than monthly consumption on the 1 (< once a month) to 7 (almost every day) scale. There were slightly more people in married or common law relationships in this cluster (75.3%) compared to the overall sample (70.8%).

#### Cluster 5: Frequent walkers with infrequent strenuous exercisers with higher alcohol use

Comprised of 13% of participants, Cluster 5 is similar to Cluster 2 with higher than average walking frequencies (*M* = 4.0; *SD* = 0.1) and lower than average strenuous exercise (*M* = 1.2; *SD* = 0.3). However, these two clusters are differentiated by alcohol consumption with the average drinking frequency for this group being 1 point away from “almost every day” on a 1–7 scale (*M* = 6.0; *SD* = 1.0). Differences in demographics also distinguish these two clusters: there were fewer people aged 45–54 in this cluster compared to overall (20.0% vs. 27.1%), more Males (55.7% vs. 49.8%), and more people in married or common law relationships (76.2% vs. 70.8%).

#### Cluster 6: Occasional and daily smokers who infrequently eat fruits and vegetables and exercise

Nearly all participants who smoked occasionally or daily were included in this cluster (8% of total). Participants in this cluster also ate, on average, 1 less serving of fruits and vegetables per week (*M* = 3.0; *SD* = 1.7) compared to the overall sample (*M* = 4.0; *SD* = 1.8). Additionally, the average level of strenuous exercise in this group was closer to “never” (*M* = 1.3; *SD* = 0.6) than the overall sample whose average was closer to “seldom (1–2 days per week”; *M* = 1.6; *SD* = 0.8). Demographically, this group was skewed towards younger age groups (e.g., 37.3% aged 45–54 vs. 27.1% final sample) and lower income brackets (e.g., 10.6% with income <$20,000 vs. 4.1% final sample). Lastly, this group was comprised of 15.7% less married or common law individuals, compared to overall, and 7.1% more single people and 5.8% more divorced participants.

#### Cluster 7: Infrequent sedentary activities

The smallest cluster by group membership (6%), people assigned to this cluster engaged in sitting activities, on average, between “seldom (1–2 days)” and “sometimes (2–4 days)” (*M* = 2.6; *SD* = 0.6) compared to the overall sample who, on average, participated in sitting activities closer to “often (5–7 days)” (*M* = 3.9; *SD* = 0.4). Demographically, this group contains 10.3% more people aged 45–54 than the overall sample.

### Partial Correlations (Baseline)

**Fig. 3.**
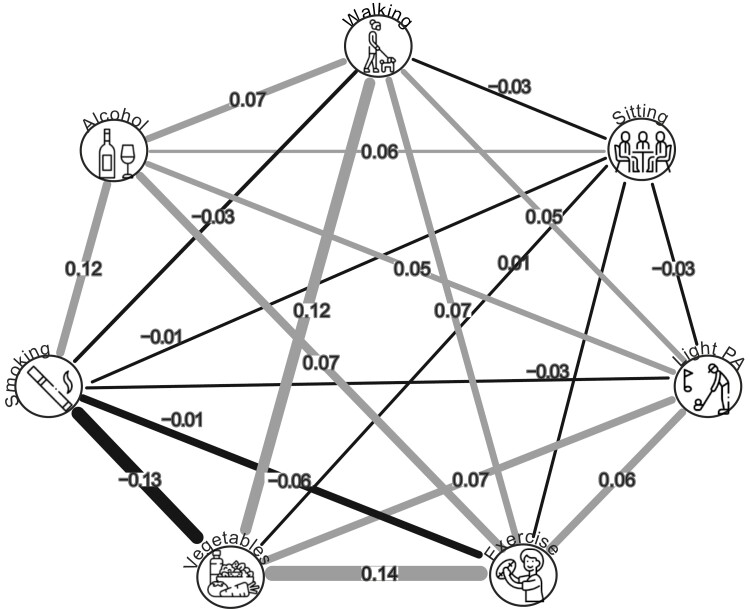
Partial polychoric correlation visualization of baseline health behaviors. Values represent partial polychoric correlations between variables. Black lines represent negative correlations and grey lines represent positive correlations. Line width corresponds to correlation strength

Partial polychoric correlations are visualized as a network in Fig. 3. Correlations ranges from ρ = −0.13 for smoking and fruit/vegetable consumption and ρ = 0.14 for exercise and fruit/vegetable consumption. The average correlation was ρ = ±0.06.

### Predicting Follow-Up Indicators from Baseline Behaviors

Two sets of exploratory regression analyses were performed with baseline health behavior clusters and individual health behaviors, predicting three health outcome indicators at follow-up. Summary statistics for health indicators are presented in Supplementary Appendix III while regression outputs are summarized in Table 3. While controlling for age and sex, clusters predicted general health *R*^2^ = 0.05, *F*(8, 37466) = 228.4, *p* < .001, mental health *R*^2^ = 0.02, *F*(8, 37485) = 78.82, *p* < .001, and chronic conditions (*AIC* = 12,629). Individual behaviors also predicted general health *R*^2^ = 0.08, *F*(9, 37465) = 368.9, *p* < 0.001, mental health *R*^2^ = 0.03, *F*(9, 37484) = 128.3, *p* < .001, and chronic conditions (*AIC* = 12,607).

**Table 3:** Regressions predicting follow-up health indicators from baseline behaviors and clusters

	General health		Mental health		Chronic conditions	
	β	*p*	β	*p*	OR	*p*
Cluster 1	0.22	<.001	0.10	<.001	0.83	<.01
Cluster 2	−0.12	<.001	−0.09	<.001	1.08	.35
Cluster 3	−0.32	<.001	−0.18	<.001	1.67	<.001
Cluster 5	0.10	<.001	0.04	.02	1.00	.97
Cluster 6	−0.44	<.001	−0.28	<.001	1.16	.15
Cluster 7	0.01	.63	−0.04	.07	0.89	.30
Age	−0.01	<.001	0.00	.01	1.09	<.001
Sex	−0.07	<.001	0.05	<.001	0.56	<.001
	*R* ^2^ = 0.05		*R* ^2^ = 0.02		*AIC* = 12,629	
Walking	0.07	<.001	0.02	<.001	0.92	<.001
Sitting	−0.03	<.01	−0.01	.46	1.16	<.01
Exercise	0.16	<.001	0.07	<.001	0.85	<.001
Light PA	0.08	<.001	0.04	<.001	0.95	.34
Fruit/Vegetable	0.05	<.001	0.04	<.001	1.01	.36
Smoking	−0.13	<.001	−0.08	<.001	1.06	.07
Alcohol	0.05	<.001	0.03	<.001	0.96	<.01
Age	−0.01	<.001	0.00	<.001	1.09	<.001
Sex	−0.05	<.001	0.08	<.001	0.58	<.001
	*R* ^2^ = 0.08		*R* ^2^ = 0.03		*AIC*= 12,607	

Cluster labels are as follows: Cluster 1 = physically active healthy eaters; Cluster 2 = frequent walkers with infrequent strenuous exercise and infrequent alcohol use; Cluster 3 = infrequent alcohol use, walking, light sports, and exercise; Cluster 4 = frequent alcohol use and infrequent walkers; Cluster 5 = frequent walkers with infrequent strenuous exercise but higher alcohol use; Cluster 6 = occasional and daily smokers who infrequently eat fruits and vegetables and exercise; Cluster 7 = infrequent sedentary activities. OR = odds ratio. All predictors listed in Table 3 are those included in the model.

## Discussion

Multiple health behaviors are robustly associated with the development of preventable non-communicable diseases and people engage in different combinations of these behaviors to varying degrees. To identify which behaviors are associated with one another to support multiple health behavior change interventions, it may help to first identify which behaviors co-occur and/or co-vary; co-occurrence and co-variation are assessed through person- and variable-centered approaches. In this study, we compared outputs from person centered (cluster analysis) and variable-centered (partial correlation) approaches. Using representative data from the CLSA, our cluster analysis produced seven groups of individuals based on similarities of frequencies they engage in key health behaviors (e.g., walking, sitting, light sports, exercise, fruit and vegetable consumption, smoking, and alcohol use). Overall, clusters were differentiated by six of the seven health behaviors included in the analysis with the most variability observed in weekly walking frequency, strenuous exercise, and alcohol consumption. Specifically, three clusters were partly characterized by walking frequency and two were characterized by strenuous exercise and alcohol consumption, respectively. Of the remaining health behaviors, there was little variability in weekly “light sports” frequencies within the seven clusters, while one cluster was generally defined by a relative extreme of a single behavior (infrequent sedentary activities). Sociodemographic characteristics varied across several clusters while associations between self-reported physical/mental health and cluster memberships were generally small.

In contrast, a partial correlation approach revealed small associations between health behaviors ranging from ranges from ρ = −0.13 for smoking and fruit/vegetable consumption and ρ = 0.14 for exercise and fruit/vegetable consumption. Minimal effect sizes of interest are not well established in the multiple health behavior change literature and it is unknown whether the small effect sizes observed in this study represent more than the “crud factor,” the idea that in the behavioral research everything correlates with everything else [40]. For example, in some fields within psychology a correlation less than ρ = 0.10 is not considered hypothesis supporting as the observed relationships between theoretically relevant and irrelevant constructs can reach this level of effect size [41].

A comparison between co-occurrence and co-variation approaches reveals strengths and limitations to each approach. Regarding limitations, neither approach modeled some known phenomena. For example, the combination of high physical activity and frequent sedentary behavior is common in individuals who participate in sports and strenuous exercise [42]; this distinction was not captured in the cluster analysis which illustrates the trade-offs between parsimony and nuance using hierarchical cluster analysis to describe co-occurring health behaviors. Additionally, the clustering algorithm revealed associations that were overlooked with variable-centered analyses. Specifically, three clusters were defined by varying combinations of walking frequency and alcohol consumption while correlations between the two variables were negligible. Taken together, these findings highlight the need for alignment between methods and research objectives with person-centered approaches more suitable for identifying sub-groups for intervention targeting purposes and variable-centered approaches more appropriate for understanding the strength and direction of relationships between interconnected behaviors.

In addition to comparing insights into health behavior associations from person- and variable-centered approaches we also investigated the ability of these approaches to predict future health outcomes. Between 2% and 8% of variability in general and mental health at follow-up were accounted for by baseline clusters or individual health behaviors. Although individual behaviors accounted for more explained variance and had better model fit, these differences were small and firm conclusions regarding the comparative utility between approaches are not yet warranted on the basis of one study but point to opportunities for future research. When classifying whether people reported any chronic conditions at follow-up, the baseline cluster of “infrequent alcohol use, walking, light sports, and exercise” was the strongest predictor of having at least one condition while the individual behavior of exercise was the strongest predictor of not having a chronic condition. To the best of our knowledge, no health behavior clustering studies in adults have produced a grouping similar to the cluster we named “infrequent alcohol use, walking, light sports, and exercise.” Behaviorally, this cluster was defined by little to no physical activity of any kind, nonsmoking, and less frequent alcohol and fruits/vegetable consumption than average. The only behavior that was above the final sample average were sedentary behaviors. People in this group tended to be older, have lower annual incomes, have higher BMI’s, use healthcare services more frequently, not be employed, and be women. Taken together, the “infrequent alcohol use, walking, light sports, and exercise” cluster may present a relatively homogenous behavioral sub-group to target for researchers and practitioners interested in conducting health behavior interventions.

This research is subject to limitations worth noting when interpreting the findings. First, many of the items selected for planned analysis are self-report which have known and inherent strengths and weaknesses [43]. Second, direct comparisons between multiple health behavior studies is difficult due to variations in sample, measurement characteristics, and inconsistent naming conventions. Although heterogeneous samples and measurement variability may be useful for establishing the presence of robust phenomena in the form of co-occurring behaviors, we encourage future analysis to clearly label clusters to include each prominent health behavior. For example, a cluster defined as “occasional and daily smokers who infrequently eat fruits and vegetables and exercise” is more clearly defined than “smokers with other risk behaviours.” Third, the health behaviors included in the cluster analysis were not exhaustive (e.g., sleep hygiene, substance use, sexual risk behaviors were not included) and some behaviors were overrepresented such as of physical activity. While grouping people based on the types of physical activities scored with the PACE scale [26] enabled us to explore variability in walking activities, the way in which physical activity frequency was measured and the nature of the categories made it difficult to evaluate the health behaviors of this sample relative to behavioral guidelines.

Although a single comparisons between methods is not definitive, person-centered approaches appear better suited than variable-centered approaches or the purposes of identifying and prioritizing targets for multiple health behavior change interventions. However, given the limitations of cluster analysis, we suggest that future person-centered research employ the “behaviour profile approach” [18] with measures linked to behavioral guidelines in order to identify all possible combinations of “meets guidelines/does not meet guidelines” for behaviors that contribute to negative health outcomes. Such approaches should ideally focus on datasets that include measures of health behavior that provide an ability to directly link behavior performance to thresholds recommended in guidelines. For variable-centered approaches, the issue of measurement heterogeneity can be addressed through the use of meta-analysis. Although meta-analytic work on the associations between health behaviors has not yet conducted, some studies are planned for the future [44].

In conclusion, the scope, size, and rigour of the CLSA dataset provided an unprecedented opportunity to investigate how health behaviors are interconnected and to compare methods for modeling this interconnectivity. Our findings show how the population of older adults in Canada can be segmented by the multiple health behaviors that characterize people’s lives and that these segmented clusters are socially patterned and associated with different health outcomes. Comparing a person- and variable-centered approach can lead to insights about behaviors that may be overlooked with a single approach. Additionally, our analyses highlights opportunity for behavioral measures to be tied to national guidelines, which could lead to even more actionable analyses. The “health behaviour profile” approach may be especially useful for future person-centered analysis, and a systematic review with meta-analysis could help establish associations between behaviors using a variable-centered approach in future research. Understanding which behaviors co-occur and co-vary, and for whom, is an important first step towards developing tailored health behavior change interventions. Future research will further develop our understanding of how interconnected health behaviors influence health outcomes over time sing longitudinal data with multiple follow-up assessments.

## Disclaimer

The opinions expressed in this manuscript are the author’s own and do not reflect the views of the Canadian Longitudinal Study on Aging.

## Supplementary Material

kaad008_suppl_Supplementary_Materials

## Data Availability

De-identified data from this study are not available in a public archive. Please contact the CLSA for data access.
